# Lymphoblastoid Cell lines: a Continuous in Vitro Source of Cells to Study Carcinogen Sensitivity and DNA Repair

**Published:** 2012

**Authors:** Tabish Hussain, Rita Mulherkar

**Affiliations:** Advanced Centre for Treatment, Research and Education in Cancer, Tata Memorial Centre, Navi Mumbai, India.

**Keywords:** Lymphoblastoid cell lines, Epstein Barr virus, cell immortalization, carcinogen sensitivity, DNA damage/repair

## Abstract

Obtaining a continuous source of normal cells or DNA from a single individual has always been a rate limiting step in biomedical research. Availability of Lymphoblastoid cell lines (LCLs) as a surrogate for isolated or cryopreserved peripheral blood lymphocytes has substantially accelerated the process of biological investigations. LCLs can be established by in vitro infection of resting B cells from peripheral blood with Epstein Barr Virus (EBV) resulting in a continuous source, bearing negligible genetic and phenotypic alterations. Being a spontaneous replicating source, LCLs fulfil the requirement of constant supply of starting material for variety of assays, sparing the need of re-sampling. There is a reason to believe that LCLs are in close resemblance with the parent lymphocytes based on the ample supporting observations from a variety of studies showing significant level of correlation at molecular and functional level. LCLs, which carry the complete set of germ line genetic material, have been instrumental in general as a source of biomolecules and a system to carry out various immunological and epidemiological studies. Furthermore, in recent times their utility for analysing the whole human genome has extensively been documented. This proves the usefulness of LCLs in various genetic and functional studies. There are a few contradictory reports that have questioned the employment of LCLs as parent surrogate. Regardless of some inherent limitations LCLs are increasingly being considered as an important resource for genetic and functional research.

Advancement in biomedical research has been spurred in part by the availability of cell lines from biological material which offer long lasting supply of cells with matching genotypes and phenotypes. One of the major contributions to this achievement among others is the ability to establish Lymphoblastoid cell lines (LCLs). LCLs are developed by infecting peripheral blood lymphocytes (PBL) with Epstein Barr Virus (EBV) which has been demonstrated to immortalize human resting B cells in vitro giving rise to an actively proliferating B cell population (1).

EBV encoded nuclear antigenic protein EBNA2 and latent infection membrane protein LMP1 play crucial role in cell immortalization along with other latent phase proteins, EBNA-1, EBNA-LP, EBNA-3A, EBNA-3C (2, 3). This method is successfully in use from last few decades with minimal amendments providing an excellent model system with various benefits as LCLs. LCLs are relatively easy to prepare and the maintenance is effortless. They also exhibit minimum somatic mutation rate in continuous culture (4).They provide an unlimited source of biomolecules like DNA, RNA or proteins and are a promising in vitro model system for genetic screening studies, genotype-phenotype correlation studies, a variety of molecular and functional assays related to immunology and cellular biology studies (5-8).

Besides this, utility of LCLs has been fully exploited mainly in studies where a single sample is required for a variety of assays. In such cases, repeated collection of the starting material - either blood or tissue from individuals becomes impractical, especially from patients who are lost to follow up or due to death of the subject during an ongoing study. Utility of LCLs in in vitro carcinogen sensitivity and DNA damage/repair studies accounts for major segment of such studies and has been very frequently documented (9-13). In almost all such reports, LCLs have proven their worth and have emerged as a promising tool. Considering enormous usefulness in all fields of biological research, LCLs have been regularly used in various studies, however, there are a couple of reports where the potential use of LCLs as a surrogate of isolated lymphocytes has been questioned and re-evaluated by comparing the results with freshly isolated or cryopreserved lymphocytes (14-17).

This assessment is important because, even though immortalised LCLs originate from normal peripheral blood lymphocytes, they do undergo significant transformation to become immortal which can alter the biology of the cell and must be taken into consideration in any analysis. Keeping all this in mind, we evaluate the promises and possible drawbacks of using lymphoblastoid cells as a surrogate for freshly prepared or cryopreserved lymphocytes and their performance and utility for in vitro carcinogenic sensitivity and DNA repair studies. The selected references and the topics that we have exemplified serve as representative illustrations covering available literature.

**Generation of lymphoblastoid cell lines**

Immortalization of mammalian cells is very frequently done by challenging primary cells with viral agents that bring about certain genetic changes making cells grow indefinitely. This transformation involves transfer of DNA tumour virus genes including Epstein Barr Virus Nuclear Antigen (EBNA), Simian virus 40 (SV40) large T antigen, adenovirus E1A and E1B, and Human Papillomavirus (HPV) E6 and E7 (18).

These agents differ in the mechanism of cell immortalization, their transformation efficiency and hence effect on the transformed cell making the selection of method of cell line preparation cell type specific. For lymphocytes, transformation by EBV is a method of choice as it imparts least genetic changes as the virus remains in the episomal form inside the host cell and only a few viral genes are expressed apparently causing minimal change in the host genome (Fig.1) (1)

**Epstein-Barr virus and Mechanism of transformation**

Epstein-Barr virus is a gamma herpes virus and causative agent of infectious mononucleosis in humans. Approximately 95% of adults are carriers of this virus having a positive antibody titre showing high persistence in the host (1). Marmoset lymphoblastoid cell line B95-8, which was established by infecting marmoset B lymphocytes with EBV isolated from human patient with infectious mononucleosis, is a constant source for producing transforming virus (1).

**Fig 1:**
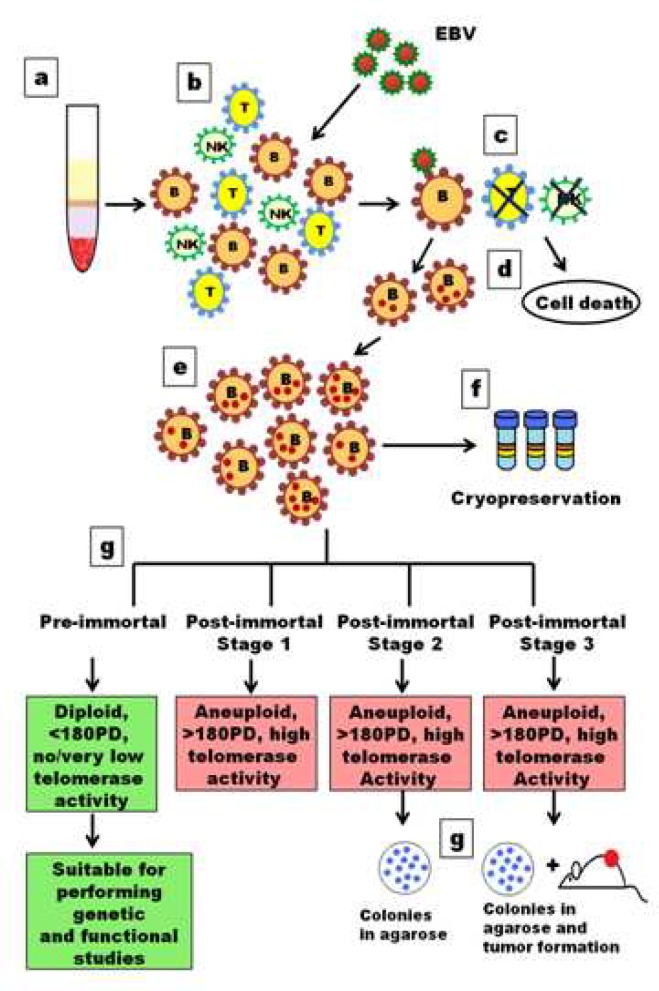
**Schematic representation of transformation of B lymphocytes with EBV** Detail of all the steps involved is explained in Table-1. Information for generating this model is taken from references 1, 3 and 6. KEY: EBV-Epstein-Barr virus, PD-population doubling, B-B cell, T-T cell, NK-Natural killer cell

The virus is known to infect only B cells in a mixed population of B, T and natural killer cells present in PBLs. The presence of complement receptor type 2, commonly known as CR2 (CD21) on B cells creates a route for virus entry into the cell (19).

Viral envelope glycoprotein gp350 binds to CR2 and triggers endocytosis (2, 20). In addition, a second glycoprotein gp42 binds to human leukocyte antigen HLA class II molecule as co-receptor (2, 21). Through these interactions, the fusion machinery is triggered and the viral membrane fuses with the endosomal membrane to release viral genetic materials inside the cell. EBV infection in other cell types, mainly epithelial cells, is less efficient and occurs through poorly defined separate pathways (21). This difference in cell tropism can be attributed, to some extent, to the type of cell from which viral preparations are made (21). For B cell immortalization, EBV establishes latent infection mainly existing as covalently closed circular episome with 5-800 copies per cell (1). This latent infection is characterized by expression of limited number of viral genes (22, 23).

The expression of these viral gene products comprises six EBV encoded nuclear antigenic proteins, EBNA1, -2, -3A, -3B, -3C, and -LP and three latent infection membrane proteins, LMP1, -2A and -2B (2-3, 24). In addition, LCLs also show abundant expression of two non-polyadenylated RNAs, EBER1 and EBER2 with unclear function (2, 3, 24). This pattern of latent EBV infection which activates only in B cell infection is referred to as latency III (2). The role of EBV latent genes has been confirmed by recombinant EBV genetic analyses with in vitro B lymphocyte transformation assays. Studies using recombinant EBV revealed indispensable requirement of EBNA2 and LMP1 for immortalization process with a crucial role of EBNA-1, EBNA-LP, EBNA-3A, EBNA-3C (2-3). 

EBNA2 acts as a transcriptional transactivator by interacting with sequence specific DNA binding protein to regulate EBV latency gene expression in B cells and to modify cellular gene expression which results in stimulation of G_0_ to G_1_ cell cycle progression resulting in B-cell immortalization (2, 25-29). Similarly, LMP1 is a critical determinant of EBV-mediated transformation as it is required for both, the outgrowth of LCLs in vitro as well as continued proliferation of an established LCLs (30).

LMP1 aggregates cellular proteins of the tumour necrosis factor receptor signalling pathway to activate transcription factor NF-κB (3). These are important events in cell immortalization as EBV recombinants with LMP1 mutations that are compromised for NF-κB activation as well as EBV strain P3HR-1 which carry deletion in gene encoding for EBNA2 are impaired for growth transformation (2-3).

**Biological characteristics of LCL**

Immunophenotyping of LCLs confirms that the cells are positive for B cell marker CD19 and negative for T cell marker CD3 as well as for NK cell marker CD56. Average population doubling time of LCLs is 24 hrs; they grow as clusters and exhibit typical rosette morphology due to the expression of adhesion molecules leukocyte function antigen 1 (LFA-1) and intercellular adhesion molecule 1 (ICAM-1) (1, 4, 31).

LCLs are commonly considered as ‘immortal cell lines’ that can grow for a large number of population doublings retaining a diploid karyotype without becoming tumorigenic (8). However, the classification of LCLs as ‘immortal cell lines’ is slightly ambiguous since most of the LCLs are mortal, named as ‘preimmortal’ LCLs because of low level of telomerase activity and shortening of telomeres with each cell division. Although few LCLs truly become immortalized, named as "post immortal" LCLs by development of strong telomerase activity and aneuploidy accompanied with other changes including down regulation and mutation of certain genes (8).

Thus LCLs are considered immortalized only if they satisfy the following criteria: (a) activation of telomerase, (b) aneuploidy and (c) exceed at least 180 population doublings (8). Even in the post-immortal phase LCLs can be further subdivided into post-immortal stage-1 (>180 PD and high telomerase activity), post-immortal stage-2 (>180 PD, high telomerase activity and ability to form colonies in agarose) and post-immortal stage-3 (>180 PD, high telomerase activity, ability to form colonies in agarose and tumour in nude mice) (Fig.1) (8).The reason for acquisition of such characters in post-immortal phase can be attributed to events like differential regulation of certain genes and instability of chromosomes (8). Hence it is recommended that LCLs be used within 2-3 months, which is far less than 180 population doublings assuming cell doubling time as 24 hr, so as to minimize the undesirable and questionable effects of post immortalization (5). 

**Advantage of using Lymphoblastoid cell lines**

It is over five decades now since the technique of animal cell culture was introduced in mid 1900s with the development of the first human cell line Hela. This has had profound benefits in biology and medicine. Cell lines have proven to be highly cost effective biological tool as compared to animal models owing to features like accelerated growth, minimal nutrient requirements and close resemblance to in vivo conditions. In addition, immortality gives main advantage for the use of cell lines for research as it can be grown indefinitely in culture. In comparison to other tissue derived adherent cell lines LCLs exhibit added advantages, first being the ease of obtaining starting material which is lymphocytes isolated from peripheral blood by a near non-invasive way. 

There is an ease of establishment of primary culture requiring no disaggregation from the primary tissue, therefore fewer chances of contamination due to reduction in the number of steps involved in establishing LCLs. In addition EBV transformed cell lines show chromosomal stability up to high population doublings in contrast to other transformed cell lines (1). The growth of LCLs in suspension and their minimal requirement for medium allows cultivation up to high cell density without much requirement of work and expense (table 1) (1). 

**Table-1:** Steps involved in establishment of Lymphoblastoid cell line with Epstein Barr virus

Isolation of peripheral blood lymphocytes	To obtain peripheral blood lymphocytes approximately 3ml of blood is taken and mixed with equal amount of 1XPBS and subjected to density gradient separation using Ficoll-hypaque.
Infection of Peripheral blood lymphocytes with EBV	Separated PBLs are a mixture of B cells, T cells and Natural Killer (NK) cells which are suspended in complete medium and are treated with EBV isolated form B95-8 cell line in 1:1 ratio. After 24hr the EBV containing medium is aspirated and cells are allowed to grow undisturbed with regular medium change as and when required.
Specific infection of B cells	EBV specifically infects B cells only owing to the presence of EBV receptor CR2 on B cell surface resulting in transformation while other cells (T cells and NK cells) die gradually during ongoing culture.
B cell transformation	EBV genome remains in the episomal form inside B cells in multiple copies and only a few viral genes are transcribed resulting in unlimited proliferation of cells.
Expansion of transformed cells	There is a considerable cell death after EBV transformation however infected cells repopulate the culture within 3-4 weeks giving rise to continuously proliferating lymphoblastoid cell lines.
Cryopreservation	As soon as LCLs are established and expanded early population doubling cells are cryopreserved for future use.
Subdivisions in transformed LCLs	I. Pre-immortal cell lines grow for <180 population doublings show normal karyotype. They have either very low or negative telomerase activity and are suitable for performing various genetic and functional studies. II. Post-immortal cell line stage-1 grow for >180 population doublings, have high telomerase activity and show aneuploidy. III. Post-immortal cell line stage-2 grow for >180 population doublings, have high telomerase activity, show aneuploidy and have ability to grow in soft agar. IV. Post-immortal cell line stage-3 or tumorigenic stage grow for >180 population doublings, have high telomerase activity, show aneuploidy, have ability to grow in soft agar and form tumour in mice.

**Utility of lymphoblastoid cell lines for genetic and functional studies**

The property of LCLs to be able to grow in continuous culture together by maintaining a close similarity to the starting parent lymphocytes has been very well exploited in various studies leading to large body of literature. The number of publications as obtained from a search of the PubMed database from within the EndNote program by the use of the search term "Lymphoblastoid cell line" (most frequently used term for EBV LCLs) is approximately 6000. But roughly 10-15% of these publications include reports that are being carried out on T cell derived or other lymphoid origin cell lines. This reduces the number to almost 5000 publications which covers virtually every aspect of research using human EBV LCLs including studies done on EBV transformed cell lines or studies done using EBV LCLs as a model system. This list of publications includes studies where LCLs have been used as a source of basic biomolecules like, DNA, including mitochondrial DNA, RNA and protein (5, 32). DNA isolated from LCLs has been widely used for mutation analysis (32-34), while RNA isolated from these cell lines has been commonly used for cDNA library preparation and to assess transcriptional response to genotoxins using high throughput technologies including cDNA microarray (35-37) together with this LCLs have as well been used for proteomic studies (38-40). In addition, in a number of other reports, LCLs have been extensively used to measure DNA damage and repair as well as apoptosis (9, 41-42). To a great extent LCLs have also been used to assess inter-individual variation in response to DNA-damaging agents and to develop correlation between DNA repair genes and repair capacity and analyzing the relationship with cancer risk (42-44). The assessment done for past 10 years reveals that approximately 65% of published reports on EBV LCLs describe usage of these cell lines as a model system while the rest, 35% are about the basic virus biology (Fig.2). 

**Fig 2:**
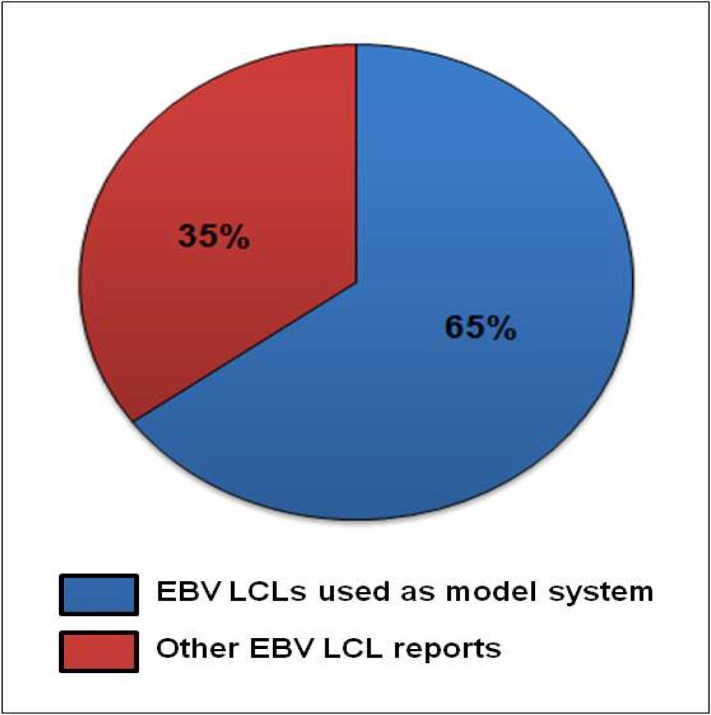
Average usage of human EBV LCLs for various studies in last one decade Percent average publications available on PubMed for last 10 years (up to 2011) for human EBV LCLs obtained from a search of the PubMed database within the EndNote program by use of the search term “lymphoblastoid cell line”. Blue region in the graph represents reports where EBV LCLs are used as a model system (65%). Red region represents other EBV LCLs reports (35%).

**Fig 3:**
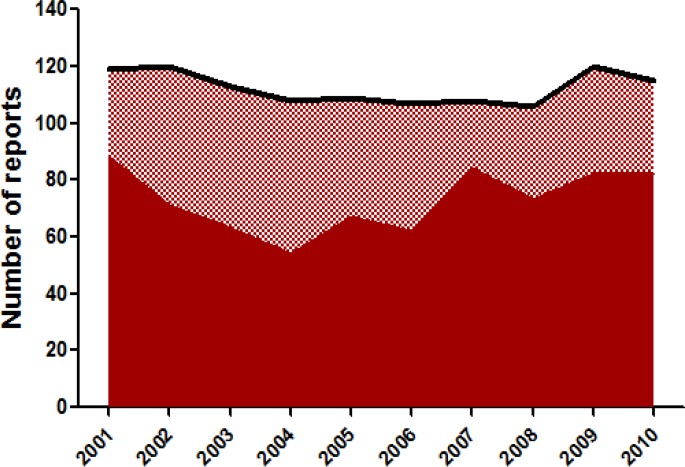
Trend of usage of human EBV LCLs in last one decade Usage of EBV LCLs for carcinogen sensitivity, DNA damage/repair and other studies has been consistent throughout the years. Solid region in the graph represents reports where EBV LCLs are used as a model system. Shaded region represents other EBV LCLs reports. Black Line above shaded region represents total available EBV LCLs reports in each year

**Fig 4:**
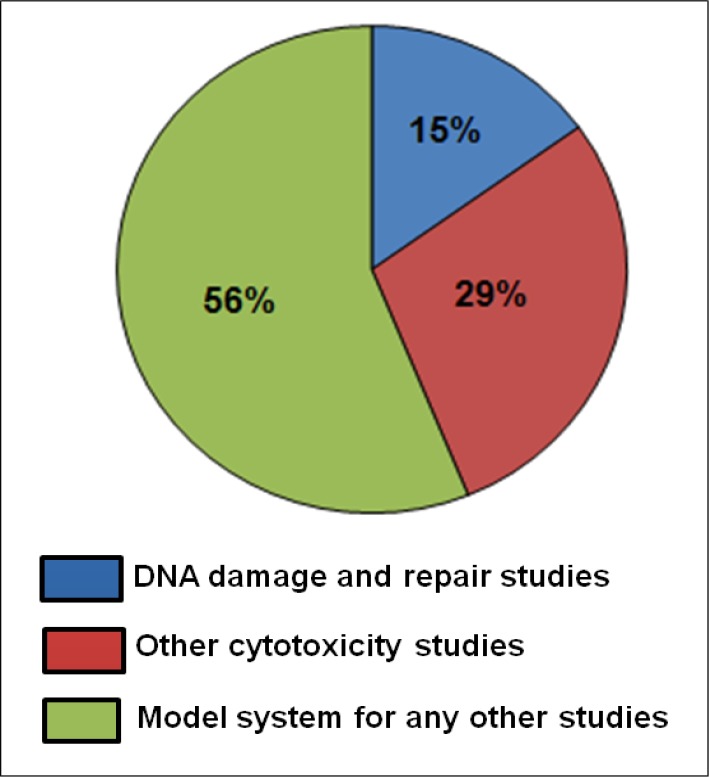
Average distribution of human EBV LCLs for various studies in last one decadeA major segment of the available reports where EBV LCLs are used as a model system comprises of cytotoxicity and DNA damage/repair studies. Blue region in the graph represents reports where EBV LCLs are used as a model system for DNA damage repair studies (15%). Red region represents other cytotoxicity studies (29%). Green region represents other studies utilising EBV LCLs model system (56%)

**Fig 5:**
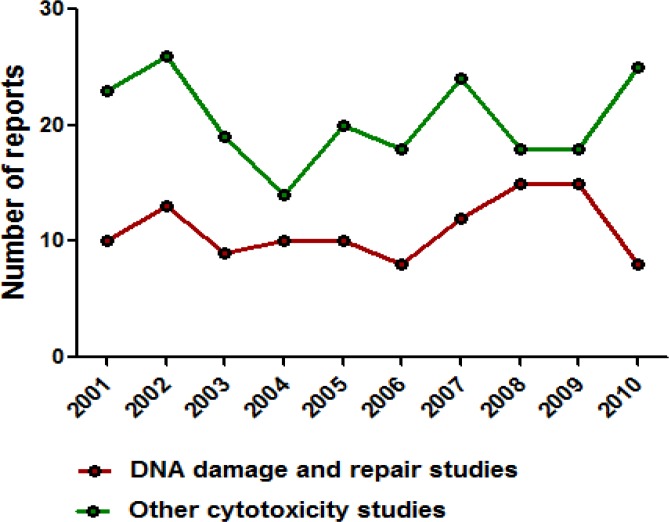
Trend of usage of human EBV LCLs as a model system for carcinogen sensitivity and DNA damage/repair studies in last one decade

 Usage of EBV LCLs as a model system for carcinogen sensitivity, DNA damage/repair and other studies has been consistent throughout

As seen from published literature, EBV LCLs have been utilized throughout the last decade, implying that these are one of the reliable and commonly used biological model systems (Fig. 3)

**Utility of lymphoblastoid cell lines for carcinogen sensitivity and DNA damage/repair studies**

Of the total reports where LCLs have been used as a model system, a major segment of 44% is represented by carcinogen sensitivity (29%) and DNA damage/repair studies (15%) (Fig.4).

The usage of LCLs in such studies over last one decade has been consistent presenting EBV LCLs as a constant and reliable source of biological material (Fig.5)

Cytotoxicity studies in present state of biomedical research include in vitro testing for drugs and patient’s sensitivity as well as carcinogenic effects of radiations or chemical compounds. A few representative examples include a recent report by Jagger et al., where they have assessed the genotoxicity of 25 pro-genotoxins on TK6 lymphoblastoid cell line as a model system using a GFP based assay (13). Similarly, Wei et al. have measured mutagen sensitivity of 16 established lymphoblastoid cell lines derived from patients with xeroderma pigmentosum, ataxia telangiectasia, head and neck cancer and melanoma as well as from normal human subjects using UV light, 4-nitroquinoline-1-oxide (4-NQO-1) and gamma radiation (45).

The efficiency of LCLs in large scale population based drug screening studies is very well shown by Persico et al., where around 70 lymphoblastoid cell lines have been used to assess the response of antiviral therapy of chronic hepatitis C virus (HCV) infection (46). Not only in basic cytotoxicity studies LCLs have proven to be an excellent model system in reports where associations are done at whole genome level (47).

A recent example is a report by Watters et al, where they have done a genome wide linkage analysis to find out loci responsible for 5-fluorouracil and docetaxel cytotoxicity using LCLs. 

arsApart from this, LCLs have also expanded their niche as a tool for a variety of DNA damage/repair studies. An excellent utilization of LCLs for such kind of research was done by Motykiewicz et al., where DNA repair capacity of 50 LCLs from sisters discordant for breast cancer was measured after benzo[a]pyrene diolepoxide exposure (BPDE) (10). Few years later the same group published a related report where they had used 300 lymphoblastoid cell lines from sisters discordant for breast cancer for measuring DNA repair (9). Subsequently the same group reported the DNA repair capacity in breast cancer patients by functionally estimating DNA repair in vitro on nuclear extracts from 179 LCLs (44). In addition, Sigurdson et al., have used LCLs for prospective analysis of DNA damage/repair markers of lung cancer risk using around 230 patients and control cell lines (48).

Not only for direct measurement of DNA repair capacity, DNA damage/repair in LCLs has been used by Schirmer et al. for identification of potential biomarker for radiosensitivity (49). Similarly, Bishay et al. have used LCLs for finding DNA damage-related gene expression as biomarkers to assess cellular response after gamma radiation exposure (50).

Although citation of all the available carcinogen sensitivity and DNA damage/repair studies where LCLs are used as a model system is not possible in this article, but one thing that is very clearly revealed is the usage of LCLs as an ideal model in place of fresh biological material to carry out any kind of experiments which is applicable to small scale studies, genome wide association studies and even large population based studies. 

**Limitations of using LCLs**

Most of the above studies show LCLs as a good surrogate to study the effect of genotoxin exposure; however, some reports do have contrary observations. Trenz et al. have demonstrated that lymphoblastoid cell lines with a BRCA1 mutation do not generally show similar chromosomal radio sensitivity by micronuclei test as lymphocytes with same BRCA1 mutation (17). Later, in 2005, they have shown that radio sensitivity of lymphoblastoid cell lines with a heterozygous BRCA1 mutation is not detected by comet assay and pulsed field gel electrophoresis (14). Similarly, in a report by Baeyens et al, it was revealed that the enhanced chromosomal radio sensitivity observed in fresh blood cultures of breast cancer patients is not present in EBV-transformed cell lines derived from the same blood samples (51).

In a recent report by Zijno et al, DNA repair capacity was compared between PBMCs from 20 healthy subjects and EBV-transformed LCLs derived from the same individuals by γH2AX foci formation assay and micronuclei test and it was observed that larger inter-experimental variability occur in the results obtained with LCLs compared to PBMCs (16). In addition Mazzie et al. have shown LCLs to be inappropriate model system for DNA repair studies with cell-free based functional assays (15).

**Probable reason for limitations**

All of the above studies have very well demonstrated the limitation of using LCLs as a surrogate of isolated lymphocytes. Interestingly such reports where the usage of LCLs as a promising surrogate of human lymphocytes is questioned account for only 2% of the total available reports where LCLs are used as model system in last one decade (Fig. 6).

One of the noticeable reasons for occurrence of such variation in observations between LCLs and lymphocytes could be the use of stimulated lymphocytes which is mainly T cell population, as this is the only subpopulation reactive to PHA, whereas LCLs are derived specifically from B lymphocytes. More over differences in the mutagenic response can occur as LCLs are exposed in the G1/S/G2 phase of the cell cycle as they grow and undergo continuous cell division whereas, stimulated T lymphocytes remain only in G0 phase and cells are known to have different sensitivity to DNA damaging agents in different phases of the cell cycle. In addition, if the assays are performed directly on the blood samples and not on isolated lymphocyte cultures, the differential response may arise due to difference in the composition and antioxidant status of the blood as compared to the medium in which LCLs are grown. Furthermore, the type of assay used to compare the sensitivity of LCLs with lymphocytes can also give rise to variation due to varied sensitivity of the assays. Therefore, care must be taken to ascertain the suitability of using LCLs in certain experiments and selecting the type of assay. 

**Fig 6:**
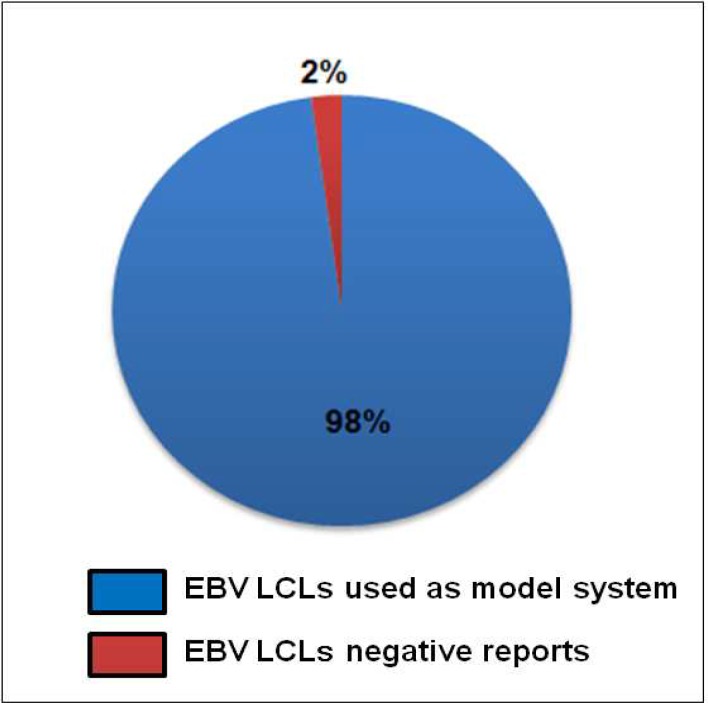
Comparison of average reports using EBV LCLs as model system with reports where EBV LCLs are not considered as a right representative for lymphocytes Blue region in the graph represents reports where EBV LCLs are used as a model system (98%). Red region represents studies where EBV LCLs are shown to be a compromising model system (2%).

**LCLs can be an ideal surrogate for lymphocytes (few other reports)**

Although there are few studies where the utility of LCLs in place of isolated lymphocytes has been shown to be compromised but at the same time there are couple of reports which provide evidence for the relevance of their use in biological research. Few examples include findings by Talebizadeh et al. where they have assessed the feasibility of using LCLs to study the role of miRNAs in the etiology of autism and arrived on a conclusion that evaluating LCLs samples is very informative in detecting changes for a subset of brain-expressed miRNAs (52). 

Similarly, to test for genotypic errors potentially caused by EBV transformation, Herbeck et al. compared single nucleotide polymorphisms (SNP) using Affymetrix GeneChip Human Mapping 500k array set in peripheral blood mononuclear cells (PBMCs) and LCLs from the same individuals and it revealed that genotypic changes found in PBMCs and LCLs were not significantly different and LCLs constitute a reliable DNA source for host genotype analysis (53).

Analogous to above report Whole-exome sequencing of DNA from peripheral blood mononuclear cells (PBMCs) and EBV transformed lymphocytes from the same donor also provide an evidence that there occur very minor changes in EBV LCLs and that too at a higher population doubling hence, LCLs are an appropriate representation of the donor (54). 

**Our experience and view on use of LCLs**

In our laboratory we have established lymphoblastoid cell lines and partially characterized them by comparing few of their phenotypic and genotypic characteristics with isolated parent lymphocytes. Common characteristics of the cell lines which include, DNA ploidy, cell surface markers, growth rate and gene expression are found to be comparable to starting lymphocytes proving them to be ideal replacement (Fig.7).

**Fig 7:**
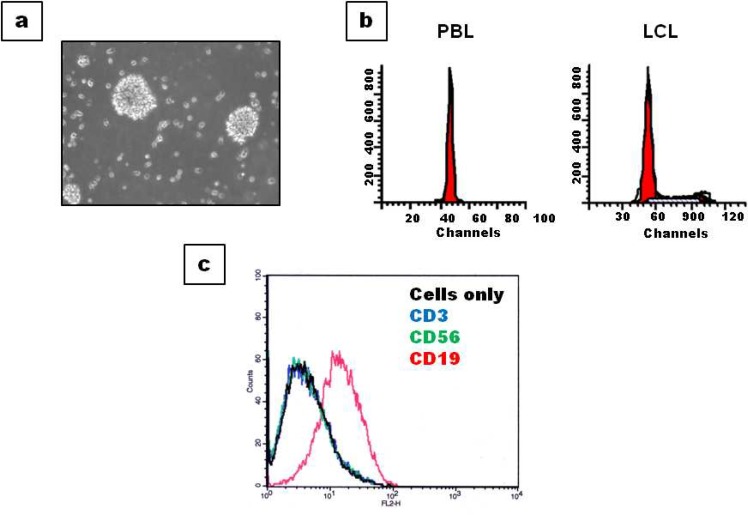
EBV LCLs Characteristics EBV cell lines generated in our lab were subjected to partial characterisation; (a) light microscopy image of LCLs showing typical rosette morphology and growth of cells in clumps at 20X; (b) DNA ploidy status of LCLs is comparable to isolated parent lymphocytes; (c) LCLs show positivity for B cell specific marker CD19 and is negative for T cell and NK cell markers CD3 and CD56 respectively

**Fig 8:**
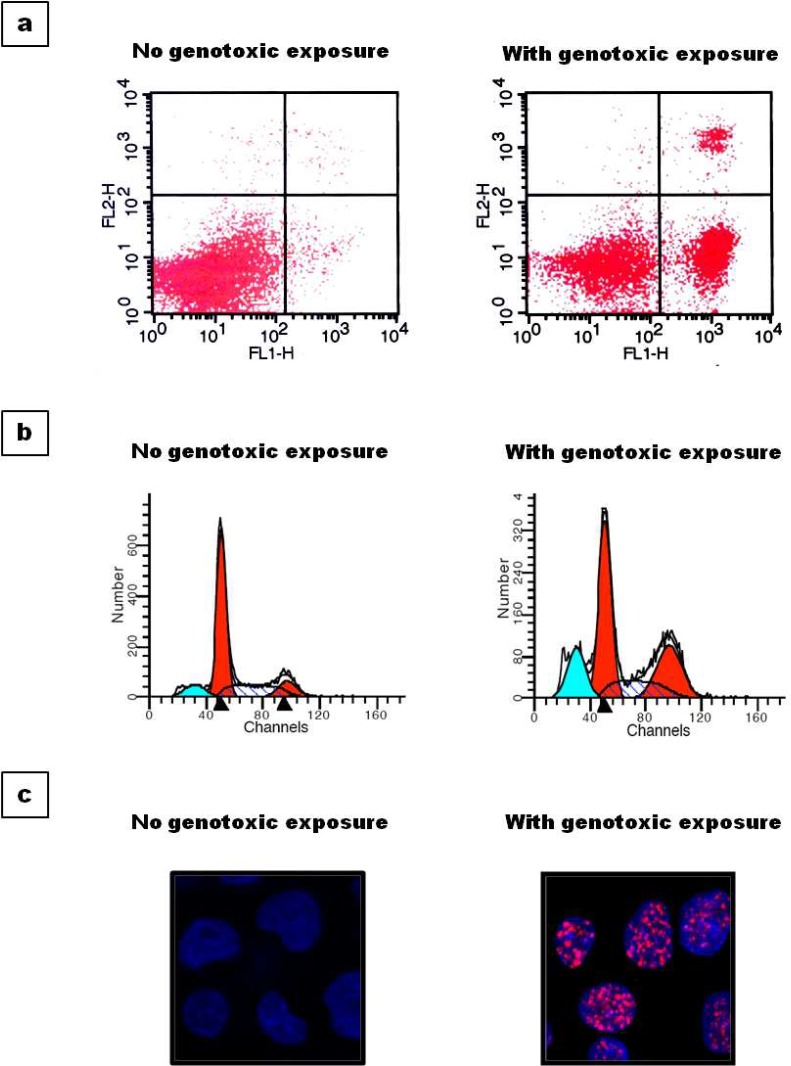
Utility of EBV LCLs for various phenotypic assays EBV cell lines generated in our lab were used for various phenotypic assays; (a) Flow cytometry data showing percent cell death; (b) cell cycle profile and (c) immunofluorescence foci formation assay after LCLs are exposed to genotoxic agent. LCLs showed expected response in all types of experiments

We have used these LCLs for performing basic in vitro phenotypic assays like assessment of DNA damage/repair and cell death as well as cell cycle profiling after genotoxic exposure along with genomic DNA isolation (Fig. 8). The results that are obtained are promising and in concert with the published data, further strengthening our belief that LCLs can be an ideal substitute of peripheral blood cells.

## Conclusions

The availability of continuously growing cell lines has markedly contributed to improvements in research and experimentation. Foremost significance of cell lines lies in their ability to grow indefinitely and to be shared between groups which lead to more productive research. EBV LCLs are one main candidate of this plethora of available cell lines. The use of LCLs in biological research, especially for studies on patient derived material, has offered many advantages, unique to EBV LCLs, including ease of obtaining starting material, handling and performing experiments and large scale cell line establishment, which has further enhanced their value over other available cell lines. 

In this article, we have discussed about promises and probable shortcomings of using lymphoblastoid cell lines in genetic and functional research. With information from the available reports LCLs present themselves as a valuable, cost effective, in vitro model system ensuring long-term, adequate starting material for current and future analysis. They are in use since the last few decades and their utility is increasingly being recognized as a surrogate for isolated lymphocytes. However, one has to be aware of the few inherent limitations of LCLs use. Finally, we conclude that LCLs very well imitate isolated lymphocytes and the advancements in the biological research will have been severely hampered and delayed without the availability of these cell lines. LCLs are here to stay as a promising human cell line model system. 
